# Understanding parental engagement in children and adolescents’ myopia prevention and control: a push-pull-mooring framework approach

**DOI:** 10.3389/fpubh.2026.1842146

**Published:** 2026-07-01

**Authors:** Jing Yan, Xianfeng Hu, Xiaowan Chen, Xiaoyan Wu, Min Li

**Affiliations:** Anhui Medical University, Hefei, China

**Keywords:** moderating effects, myopia prevention and control, parents, push-pull-mooring framework, willingness to engage

## Abstract

**Objective:**

Successful endeavors in myopia prevention and control require parental participation. This study applied the Push-Pull-Mooring (PPM) framework to explore how push, pull, and mooring factors foster parents’ willingness to engage in myopia prevention and control, and to examine the moderating role of information provision on the relationship between parental willingness and actual engagement.

**Methods:**

A cross-sectional study was conducted among parents of children and adolescents in the Yangtze River Delta region of China. A total of 996 participants completed a web-based self-administered questionnaire. Structural equation modeling and hierarchical regression were employed to investigate the determinants of parental willingness and actual engagement in myopia prevention and control.

**Results:**

Push factors, including perceived risk of myopia (*β* = 0.119, *p* < 0.001) and knowledge of myopia (*β* = 0.221, *p* < 0.001), was significantly associated with parental willingness to engage. Pull factors, including recognition of policies and campaigns (*β* = 0.268, *p* < 0.001) and peer influence (*β* = 0.288, *p* < 0.001), are also positively associated with willingness. Among mooring factors, cost of myopia prevention and control (*β* = −0.082, *p* < 0.01) was negatively associated with parental willingness, and also weakened the positive association with push and pull factors on willingness. Parental willingness (*β* = 0.531, *p* < 0.001) is positively associated with actual engagement; however, information provision did not significantly moderate the relationship between willingness and actual engagement.

**Conclusion:**

The PPM framework effectively explained the psychosocial determinants of parental engagement in myopia prevention and control. Peer influence and recognition of myopia prevention policies and campaigns were the strongest predictors of parental willingness, while prevention costs served as a key barrier. Parental willingness, in turn, was a strong predictor of actual engagement behavior. These findings suggest that policymakers should strengthen parental willingness through targeted campaigns that leverage peer influence, improve policy awareness, and reduce prevention-related costs through resource provision at the family level.

## Introduction

High myopia can lead to severe ocular pathologies such as myopic macular degeneration and retinal detachment, imposing substantial burdens on individuals, families, and healthcare systems (1). The global prevalence of myopia has been steadily rising, with a particularly concerning increase among children in China over the past decade (1, 2). Myopia imposes a formidable burden on public health and the economy, with recent urban-only cost estimates reaching US$26.3 billion, or 0.23% of China’s GDP (3). To address this crisis, the Chinese government has formulated and implemented several policies since 2018 to reduce myopia prevalence and improve control measures among children and adolescents (4). Unfortunately, these policies and actions have shown limited impact (5).

Studies have indicated that increasing outdoor exposure, along with environmental interventions including optimized ambient lighting and ergonomically appropriate postural support systems can effectively prevent and control myopia in children (5, 6). While schools have made efforts to increase outdoor activities on campus, extending these efforts beyond school is also important (7, 8). Such endeavors require parental involvement, which highlights the crucial role of parents in effectively preventing and controlling myopia (8–10). The success of paediatric interventions requiring behavioral change or novel treatment uptake hinges on parental recognition and acceptance of their necessity (11).

Prior research has linked perceived severity, barriers, attitudes, subjective norms, and behavioral control to parental myopia prevention intentions (7). Although parents with risk awareness adopt protective behaviors, significant knowledge gaps regarding myopia etiology and evidence-based prevention strategies remain prevalent (12). These knowledge deficits, combined with misconceptions about myopia development and the home environment parents create, profoundly impact children’s visual health trajectories (13). Most critically, the tendency to prioritize educational achievement over ocular health creates a fundamental tension that undermines vision health initiatives, suggesting that effective interventions must reconcile rather than oppose these competing priorities to advance comprehensive myopia prevention programs (14). Research has predominantly examined parental barriers and facilitators in parents’ myopia prevention. However, limited attention has been paid to the mechanisms underlying the willingness-behavior gap, particularly how motivational factors and contextual barriers interact to influence the translation of parental willingness into actual myopia control practices.

To address this gap, this study adopted the push-pull-mooring (PPM) framework to examine the underlying mechanisms shaping parental engagement in myopia prevention and control. The PPM framework originated from migration theory in demography and geography (15), where push factors drive individuals away from their current state, pull factors attract them toward alternatives, and mooring factors act as facilitating or constraining conditions that influence the transition process. The framework has since been widely applied in consumer switching behavior research and has recently been extended to health behavior and public policy contexts (16, 17). In the context of this study, the PPM framework is applied to conceptualize parental behavioral change as a switching process from non-engagement to active engagement in myopia prevention and control for children and adolescents.

Compared with other health behavior theories such as the Health Belief Model (HBM) and the Theory of Planned Behavior (TPB), the PPM framework offers distinct analytical advantages for the present study. HBM and TPB primarily focus on individual cognitive factors, such as perceived severity, attitudes, and subjective norms, in predicting behavioral intentions. However, parental engagement in myopia prevention is not solely driven by individual cognition; it is also shaped by external policy environments that attract participation and by structural barriers that constrain behavioral change. The PPM framework integrates these three dimensions simultaneously: internal motivations that push parents to consider action (e.g., perceived risk and knowledge of myopia), external incentives that pull parents toward engagement (e.g., policy campaigns and peer influence), and contextual constraints that moor or anchor existing behavioral patterns (e.g., prevention costs). This multi-dimensional structure makes PPM particularly suited to analyzing behavioral changes occurring within a policy-driven context, where individual motivations, institutional incentives, and practical barriers coexist and interact. Furthermore, willingness alone does not always translate into behavioral change, as external obstacles can deter action (18). Some research has proposed that providing information about specific behavioral changes can effectively motivate individuals to modify their behavior (19). In this regard, providing health policy-related information may help bridge the gap between willingness and actual behavior (20, 21).

Building on the PPM framework and extensive research on information provision’s effect on the relationship between individuals’ willingness to change and actual behavioral change, this study aimed to comprehensively examine how push (i.e., perceived risk and knowledge of myopia; knowledge of myopia), pull (i.e., recognition of myopia prevention and control policies and campaigns; peer influence), and mooring factors (i.e., myopia prevention and control costs) are related to parental willingness to engage in myopia prevention and control for children and adolescents. This study also investigated the moderating impact of policy-related information supply on the association between parental intention and actual engagement in myopia prevention and control.

### Push factors and parents’ switching willingness

According to the PPM theory, push factors are described as negative attributes related to the current state that prompt individuals to seek change. In the context of parental myopia prevention, perceived risk of myopia and knowledge of myopia function as push factors because they prompt parents to recognize the inadequacy of their current behaviors and motivate them to seek change (22). When parents recognize the risks of myopia and understand its causes and warning signs, they become aware of the inadequacy of their existing behaviors, which drives them to seek active engagement in prevention (23). Specifically, the perception of myopia risk refers to the extent to which parents perceive the risks or harms that myopia poses to their children. Individuals’ assessment of risks prompts the generation of coping behaviors (24). For instance, when parents have a stronger perception of disease risks, they are more willing to have their children vaccinated (25).

In addition, knowledge of myopia serves as a push factor because it enables parents to recognize the gap between their current behaviors and evidence-based prevention practices. Unlike the broader concept of health literacy, which encompasses the knowledge, motivation, and competences to access, understand, and apply health information (26), myopia knowledge in this study specifically concerns parents’ familiarity with myopia types, prevention methods, and early warning signs. When parents understand that certain daily habits, such as insufficient outdoor activity, contribute to myopia development, and are familiar with effective prevention methods and early warning signs, this knowledge creates a sense of urgency that pushes them away from their current inaction. Previous research has demonstrated that parents with better knowledge of myopia are more likely to adopt protective behaviors such as regulating near work duration and ensuring regular breaks (12).

Given that both risk perception and myopia knowledge function as internal cognitive drivers that motivate parents to move away from inadequate prevention practices, they are conceptualized as push factors in this study. The following hypotheses are proposed:

*H1:* Parental perceived risk of myopia plays a positive role in fostering parents’ willingness to switch to myopia prevention and control.

*H2:* Parental knowledge of myopia is positively associated with parents’ willingness to switch to myopia prevention and control.

### Pull factors and parents’ switching willingness

Pull factors are considered to be positive attributes that encourage the adoption of myopia prevention and control measures, specifically attracting parents to take proactive actions in controlling myopia. In this study, pull factors serve as the positive attractiveness for engaging in myopia prevention and control, pulling parents to develop the willingness to control their myopia. The leading role played by government policies on myopia prevention and control, as well as the influence exerted by fellow parents, relatives, and friends who engage in visual health protection behaviors, may contribute to motivating parents to develop a willingness to control myopia.

Myopia prevention and control policies and campaigns aim to motivate schools, parents, and other stakeholders to engage in comprehensive vision protection activities. Unlike mere information exposure, government policies provide structured incentives, institutional resources, and normative direction that actively attract parents toward preventive engagement. For instance, policies may establish vision screening programs in schools or set specific targets for outdoor activity time, creating an enabling environment that pulls parents toward participation. Divergent public understanding of government policies can lead to performance losses and social conflicts, hindering policy implementation and the achievement of effective policy outcomes (27). When parents recognize and understand these policies, they are attracted by the institutional support and practical guidance that policies provide, thereby developing willingness to engage in preventive action (28). Hence, the following hypothesis is introduced:

*H3:* Myopia prevention and control policies and campaigns is positively associated with parents’ willingness to shift to myopia prevention and control.

Beyond formal policy influences, peer influence functions as a social environmental pull factor. Peer influence exerts strong social pressure and motivation on individuals, positively impacting their willingness to adopt new behaviors and take corresponding actions (30). In Chinese family culture, parental comparison of children with their peers has become a pervasive daily practice (29). Moreover, this comparison culture extends beyond children to parents themselves, who inevitably compare their own parenting behaviors with those of their social circles. When parents observe vision protection behaviors among relatives or friends, they are prompted to evaluate and potentially adjust their own myopia control practices. Following these observations, we hypothesize:

*H4:* Peer influence is positively associated with parents’ willingness to shift to myopia prevention and control.

### Mooring factor and parents’ willingness

Mooring factors that affect individuals’ behavioral switching include situational, individual, and environmental factors. Mooring factors can either facilitate or hinder behavioral shift, with most scholarly attention directed toward impediments to behavioral change (18). Consistent with this focus, we conceptualize mooring factors as various cost barriers that may constrain parents’ ability to implement myopia prevention and control measures for their children. While the original PPM framework conceptualizes mooring factors broadly to include habitual inertia and interpersonal ties, the present study focuses specifically on cost barriers, encompassing both economic expenses and time and energy investment, given their consistent identification as primary constraints on parental engagement in myopia prevention (14). Building on this perspective, parents in our study context may perceive myopia prevention and control as time-intensive, requiring regular outdoor activities with children, frequent vision screenings, and continuous monitoring of children’s visual habits and posture. These temporal demands represent significant implementation costs that potentially diminish parental willingness to participate in myopia control. Therefore, the following hypothesis is presented:

*H5:* Myopia prevention and control costs are negatively associated with parents’ willingness to shift to myopia prevention and control.

Mooring factors not only directly influence individuals’ behavioral decision-making willingness but also modulate the impact of propulsive and attractive forces on the process of behavioral decision-making (18). Accordingly, this study proposes that mooring factors moderate the relationship between push-pull antecedents and parental switching willingness. Despite high risk perception and policy awareness, parents may resist implementing prevention behaviors when facing substantial time and effort requirements that exceed their available resources. Following these arguments, we hypothesize:

*H6:* Myopia prevention and control costs negatively moderate the associations between push factors, including (a) parental perceived risk of myopia and (b) parental knowledge of myopia on parents’ willingness.

*H7:* Myopia prevention and control costs negatively moderate the associations between pull factors, including (a) myopia prevention and control policies and campaigns and (b) peer influence on parents’ willingness.

### The shifting willingness-action gap

Behavioral willingness refers to the individual’s decision-making process regarding whether to adopt certain future behaviors or the likelihood of their adoption of specific behaviors (30). When the circumstances favor the behavior, the individual’s willingness to perform the behavior is high, making it more likely for them to carry out the actual behavior (31). That is to say behavioral willingness has a significant and direct impact on actual behavior. In this study, parental willingness towards the myopia prevention and control will facilitate the actual adoption of myopia prevention behaviors. Therefore, the present research proposes the following hypothesis:

*H8:* Parents’ willingness is positively associated with the actual switching behavior.

Furthermore, it is important to acknowledge that there is often a discrepancy between willingness and actual behavior, and that information provision can bridge this gap (32). While most parents express willingness to proactively protect their children’s visual health, lack of information on myopia prevention and control methods creates a disparity between this willingness and actual behavior (14). In this study, information provision measures parents’ perceived value of receiving myopia prevention information from multiple channels. Following the approach of Wang et al. (18), information provision scenarios were used to assess the potential moderating role of information supply on the willingness-action relationship. This approach is particularly relevant in the Chinese context, where myopia prevention policies, services, and information dissemination vary considerably across regions (33). Following these statements, we hypothesize:

*H9:* Information provision positively moderates the association between switching willingness and actual switching behavior.

## Method

### Study design and population

This cross-sectional investigation was carried out in the The Chinese Yangtze River Delta region, which includes the provinces of Anhui, Jiangsu, Zhejiang and Shanghai. This region was selected because of its implementation of numerous pilot and demonstration projects for the prevention and control of myopia. A questionnaire targeting parents of children from elementary to middle school was designed using WJX, the largest and most widely used online survey platform in China. The study made use of WJX’s paid sampling service, which includes 2.6 million registered members with diverse demographic characteristics, to guarantee data quality (24). Eligible participants were recruited from among registered WJX members, and data were collected from January to April 2025. The following were the requirements for inclusion: (1) parents of children aged 8–15 years, (2) providing informed consent and voluntarily participating in the survey, and (3) residing in the Yangtze River Delta region. A total of 172 questionnaires were excluded based on the following criteria: (1) uniform response across all questions and (2) missing data. To minimize potential common method bias, respondent anonymity was ensured and the order of questionnaire items was randomized. 996 valid questionnaires were obtained after exclusion, resulting in an 85.3% response rate.

### Measurements

The measurement items (Table 1) used in this study were modified from previous studies and improved in accordance with the PPM framework and pertinent literature to fit the current study’s setting. A five-point Likert scale, with 1 denoting ‘strongly disagree’ and 5 denoting ‘strongly agree’, was used to score each item. To assess the clarity and practicality of these measurement items, 10 students and 5 academic scholars participated in a pilot study. Their feedback was used to refine wording, correct minor spelling errors, and adjust item order.

**Table 1:** Constructs and measurement items.

Construct	Measurement items
Perceived risk of myopia	Myopia is detrimental to the healthy development of my children.
	Myopia can increase future economic costs, including myopia correction surgeries and regular glasses replacement.
	Myopia can cause inconveniences in children’s daily lives.
	Myopia can affect children’s academic performance.
Knowledge of myopia	I am familiar with the distinction between pseudo myopia and true myopia.
	I am familiar with the methods to prevent the progression of myopia.
	I am familiar with the warning signs of myopia in adolescents, including blurry vision and frequent eye rubbing.
Recognition of myopia prevention and control policies and campaigns	The implementation of policy will help prevent and control my children’s myopia.
	I am aware that the government has launched pilot projects for myopia prevention and control in various regions.
	I am aware that schools and medical institutions widely conduct myopia prevention and control campaigns.
	I am aware that myopia prevention campaigns for adolescents require eyewear retailers to ensure proper fitting and quality of eyeglasses.
Peer influence	Peers place considerable importance on protecting their children’s eyes.
	Peers encourage their children to engage in outdoor activities.
	Peers can lead by example through limiting time spent on electronic devices.
	Peers take their children to professional medical institutions for regular eye tests.
Myopia prevention and control costs	Regular eye tests require a significant amount of time and money.
	Regularly replacing glasses requires excessive time and money.
	Installing eyesight-friendly lighting and seating fixtures at home requires excessive time and money.
	Facilitating children’s participation in outdoor activities requires excessive time and money.
Information provision	If the school sets more myopia prevention requirements, I will be more willing to cooperate.
	If the community regularly provides myopia prevention information, I will be more willing to take action to protect my children’s vision.
	If the media provides more videos on myopia prevention, I will pay closer attention.
	If the government offers more expert lectures and free vision screenings for myopia prevention, I will be more motivated to engage.
	If hospitals provide more detailed, professional information on myopia prevention, I will be more motivated to engage.
Willingness to engage	I am willing to frequently remind my children about their posture and reading position.
	I am willing to ensure my children get ample rest and sufficient outdoor activity.
	I am willing to adjust the lighting and seating at home to be more eyesight-friendly.
	I am willing to reduce my screen time to set a positive example for my children.
Actual engagement	Over the past three months, I have consistently reminded my children about their posture.
	Over the past three months, I have ensured my children get ample rest and sufficient outdoor activity.
	Over the past three months, I have installed eyesight-friendly lighting and seating at home.
	Over the past three months, I have reduced my device use and screen time to set a positive example for my children.

Perceived risk of myopia was measured using four items sourced from the studies of Yuan et al. (34) and Nguyen et al. (35). Knowledge of myopia was assessed with three items developed based on the research of Li et al. (36) and Nayak et al. (37). Recognition of myopia prevention and control policies and campaigns was evaluated using four items developed according to the studies of Fan and Zhang (38) and Nguyen et al. (35). Peer influence was measured with four items referenced from the works of Lai et al. (39) and Liu et al. (40). Myopia prevention and control costs were assessed using four items adapted from the research of Liu et al. (40) and Nguyen et al. (35). Information provision was measured with five items sourced from the studies of Grant et al. (41) and Wang et al. (18). Willingness to engage was assessed using four items adopted based on the research of Fan and Zhang (38) and Liu et al. (40).

Finally, actual engagement was measured with four items referenced from the studies of Jia et al. (42) and Nayak et al. (37). As parental engagement in myopia prevention primarily occurs in home settings, this variable was assessed through parents’ self-reported behaviors over the past three months, consistent with the approach adopted in similar studies on parental myopia prevention behaviors (43). Prior to the formal survey, a pilot study was conducted to assess the clarity and contextual appropriateness of all measurement items. Ten graduate students and five academic experts were invited to review the questionnaire and provide feedback on item wording and relevance. Based on their suggestions, minor revisions were made to ensure the items were suitable for the present study context.

### Statistical analysis

AMOS 26.0 and SPSS 26.0 were used for data analysis. First, structural equation modeling (SEM) was used to examine the relationships between the research constructs. This process involved measurement model testing to assess the validity of the measurement instruments, and the proposed correlations were then investigated using structural model analysis. Second, moderating effects were tested using hierarchical regression analysis. Interaction terms were incorporated into the models to explore how specific variables influenced the relationships between other variables.

## Results

Table 2 displays the demographic details of the participants. The sample distribution across the Yangtze River Delta region was as follows: Shanghai (311), Jiangsu (290), Zhejiang (222), and Anhui (173). Among the participants, 421 were male and 575 female, and approximately 81.5% were aged between 20 and 39 years.

**Table 2:** Participant demographic characteristics (*n* = 996).

Variable	Classification	*n*	Percentage (%)
Region	Shanghai	311	31.2
	Jiangsu	290	29.1
	Zhejiang	222	22.3
	Anhui	173	17.4
Gender	Male	421	42.3
	Female	575	57.7
Age (years)	20–29	71	7.1
	30–39	741	74.4
	40–49	178	17.9
	Above 50	6	0.6
Myopia condition	Neither my spouse nor I have myopia	251	25.2
	Both my spouse and I have myopia	243	24.4
	Only I have myopia	348	34.9
	Only my spouse has myopia	154	15.5
Residence	Urban area	859	86.2
	Rural area	23	2.3
	Township	113	11.3
	Others	1	0.1
Educational level	High school or below	169	17.0
	Bachelor’s degree or above	827	83.0
Annual income	Less than 30,000 RMB	8	0.8
	30,001–50,000 RMB	20	2.0
	50,001–80,000 RMB	43	4.3
	80,001–120,000 RMB	198	19.9
	Above 120,000 RMB	727	73.0
Number of children	1	625	62.8
	2	365	36.6
	3 or more	6	0.6

Prior to data analysis, Harman’s single-factor test was conducted to assess common method bias. All items were entered into an exploratory factor analysis with principal component extraction and unrotated solution. Eight factors emerged with eigenvalues greater than 1.0, and the first factor accounted for 32.31% of the total variance, which is below the commonly used 50% threshold, indicating that common method bias is not a serve problem in this study.

### Measurement model testing

AMOS 26.0 was utilized to assess the measurement model. The fit indices indicated a good model fit: *χ*^2^/df = 1.038, CFI = 0.999; GFI = 0.973; NFI = 0.978; IFI = 0.999; the measurement model correctly reflected the dataset, as evidenced by the TLI of 0.999 and the RMSEA of 0.006 values (44).

Construct reliability was evaluated using Cronbach’s alpha and composite reliability values. Table 3 demonstrates that Cronbach’s alpha values, which ranged from 0.817 to 0.919, were higher than the widely accepted limit of 0.7. Meanwhile, internal consistency and dependability were further confirmed by composite reliability values, which varied from 0.843 to 0.938 (45).

**Table 3:** Confirmatory factor analysis results.

Construct	No. of item	Factor loading	Cronbach’s alpha	AVE	CR	PRM	KM	PC	PI	MC	WE	AE
PRM	4	0.806–0.824	0.894	0.678	0.887	**0.823**						
KM	3	0.807–0.818	0.864	0.679	0.853	0.321**	**0.824**					
PC	4	0.802–0.830	0.898	0.687	0.886	0.363**	0.379**	**0.829**				
PI	4	0.806–0.821	0.896	0.682	0.889	0.377**	0.347**	0.340**	**0.826**			
MC	4	0.874–0.895	0.919	0.700	0.938	−0.160**	−0.082**	−0.065**	−0.071**	**0.860**		
WE	4	0.774–0.807	0.903	0.699	0.865	0.390**	0.435**	0.467**	0.474*	−0.144**	**0.836**	
AE	4	0.740–0.760	0.817	0.527	0.843	0.352**	0.327**	0.344**	0.343**	−0.161**	0.434**	**0.726**

PRM, perceived risk of myopia; KM, knowledge of myopia; PC, myopia prevention and control policies and campaigns; PI, peer influence; MC, myopia prevention and control costs; WE, willingness to engage; AE, actual engagement. AVE, average variance extracted; CR, composite reliability; the square root of the average variance derived for the constructs is represented by bolded figures on the diagonal. **p < 0.01; *p < 0.05.

The average variance extracted (AVE) and factor loadings were analyzed in order to examine convergent validity. Good convergent validity was shown by factor loadings ranging from 0.740 to 0.896 and AVE values over 0.50 for all seven constructs. By contrasting the square roots of the AVEs with the correlations between the constructs, discriminant validity was investigated (46). The discriminant validity criteria was satisfied when the square roots of the AVEs were higher than the inter-construct correlations.

### Structural equation model analysis

**Figure 1:**
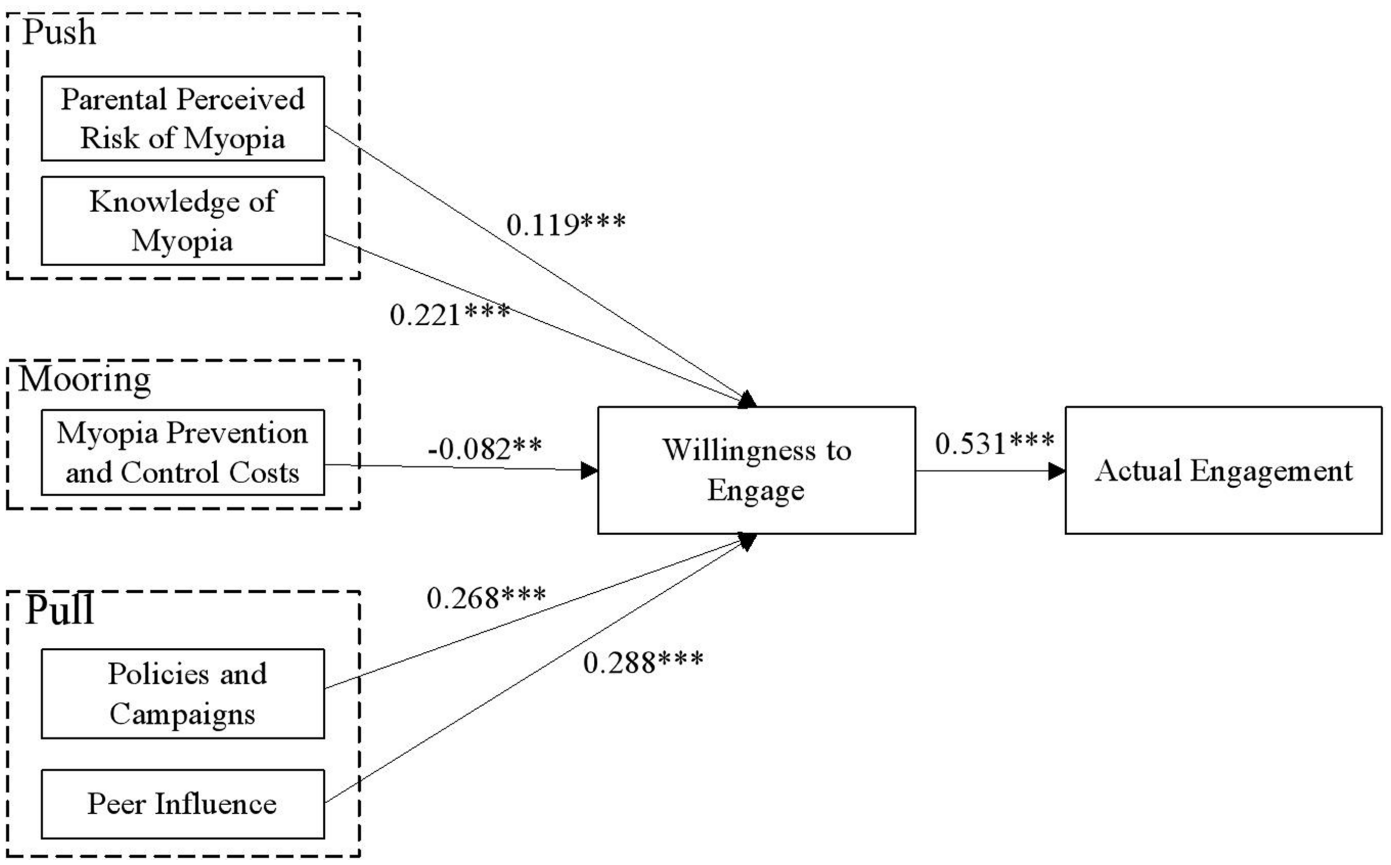
Final structural equation model of the study. ** indicates *p* < 0.01 and *** indicates *p* < 0.001.

To investigate the path linkages among the latent variables, parameter estimation was carried out using the maximum likelihood estimation approach. Figure 1 illustrates the structural model and the results of the hypothesis testing. The results showed that perceived risk of myopia (*β* = 0.119, *p* < 0.001), knowledge of myopia (*β* = 0.221, *p* < 0.001), peer influence (*β* = 0.288, *p* < 0.001), and parents’ desire to participate was positively and strongly correlated with their knowledge of myopia prevention and control policies and campaigns (*β* = 0.268, *p* < 0.001), supporting H1, H2, H3, and H4. Additionally, Parents’ willingness to participate was adversely and strongly correlated with myopia prevention and control expenses (*β* = −0.082, *p* < 0.01), confirming H5. Finally, parents’ willingness to engage was positively linked with actual engagement (*β* = 0.531, *p* < 0.001), supporting H8.

### Moderating effects analysis

SPSS 26.0 was used to analyze the moderating effects. Table 4 summarizes the findings, showing significant two-way interaction terms at *p* < 0.01 and *p* < 0.05 levels. The interaction terms between the mooring factor and the two push factors (knowledge of myopia and perceived risk of myopia) had significant negative coefficients, suggesting that myopia prevention and control costs attenuated the relationship between these push factors on willingness to engage, supporting H6a and H6b. Additionally significant were the coefficients of the interaction terms between the mooring factor and the two pull factors (peer influence and parental awareness of myopia prevention and control policies and campaigns), supporting H7a and H7b.

**Table 4:** Results of moderating effect analysis.

Moderating variable	*Β* value	*p*	Significance
PRM*MC → WE	−0.068	0.012	Significant
KM*MC → WE	−0.086	0.002	Significant
PC*MC → WE	−0.061	0.029	Significant
PI*MC → WE	−0.074	0.007	Significant

*p < 0.05; **p < 0.01.

We also looked at the moderating influence of information provision. The regression model was gradually expanded to include control factors, independent variables, moderator variables, and interaction terms. Prior to developing the interaction terms, the independent and moderator variables were mean-centered. The results are presented in Table 5. Model 1 showed that among the control variables, only residence had a significant effect on willingness to engage, whereas gender, age, and education had no significant effect. Compared to Model 1, the coefficient of determination (*R*^2^) significantly increased in Models 2 and 3, indicating that parents’ willingness to engage (*β* = 0.723, *p* < 0.001) was significantly related to their actual behavior, reaffirming H8. Information provision was also significantly linked to actual engagement (*β* = 0.497, *p* < 0.001). However, there was no significant relationship between actual involvement and the interaction term between willingness and information provision (*β* = −0.0332, *p* > 0.05). These results suggest that information provision does not moderate the relationship between willingness to engage and actual engagement. Thus, H9 was not supported.

**Table 5:** Moderating effect of information provision.

Variable	Model 1	Model 2	Model 3	Model 4
Step 1. Control variable				
Age	0.001	0	−0.001	−0.001
Gender	−0.011	−0.013	−0.021	−0.024
Myopia condition	0.004	−0.005	−0.008	−0.009
Residence	−0.085**	−0.022	−0.042**	−0.043**
Education	0.017	0.01	0.017**	0.015**
Income	−0.01	−0.002	0.003	0.001
Occupation	0.036	0.015	0.013	0.015
Number of children	−0.003	−0.028	−0.033	−0.029
Step 2. Independent variable				
Willingness to engage (WE)		0.723***	0.39***	0.347***
Step 3. Moderator variable				
Information provision (IP)			0.497***	0.455***
Step 4. Interactions				
WE*IP				−0.0332
*R* ^2^	0.011	0.545	0.626	0.628
Adjusted *R*^2^	0.003	0.541	0.622	0.624
*F* change	1.387	1157.615***	211.865***	7.2***

*p < 0.05; **p < 0.01; ***p < 0.001.

## Discussion

Parental engagement in myopia prevention and control is influenced by a highly complex interplay of factors. This study applied the PPM framework to explore the determinants of parental willingness to engage and actual engagement in myopia prevention and control. The results indicated that pull factors, push factors and the mooring factor played significant roles in shaping parental behavior, confirming the applicability of the PPM framework in this context.

The results of this study showed that the two push factors, perceived risk of myopia and knowledge of myopia, were both positively associated with parents’ willingness to engage in prevention and control. Particularly, knowledge of myopia had a stronger relationship than perceived risk of myopia. This pattern may reflect the current epidemiological context in China, where public awareness of myopia as a prevalent condition is already relatively high, yet many parents remain uncertain about the specific mechanisms of myopia development and the efficacy of preventive measures. In this context, possessing accurate and comprehensive knowledge may more directly translate into behavioral motivation, as it equips parents with the confidence and competence to act. This finding is consistent with prior PPM-based research on health-related behavior change (47, 48). It also aligns with studies in the myopia prevention literature demonstrating that parental health literacy is a significant determinant of engagement in children’s eye care (21, 41).

The results also revealed that the two pull factors, specifically the recognition of myopia prevention and control policies and campaigns and the influence from peers, were positively associated with parents’ willingness to engage in myopia prevention and control. These pull factors demonstrated a stronger relationship with willingness to engage than the push factors. This finding may reflect the broader social and institutional context in China, where government-led public health campaigns and collective social norms exert considerable influence on individual health behaviors. Given this, governments should take an active role in strengthening myopia prevention campaigns by not only expanding policy coverage but also incorporating interactive and community-based elements that facilitate peer communication among parents, as peer influence emerged as the strongest predictor of parental willingness in this study.

Furthermore, peer influence emerged as the strongest predictor of parental willingness to engage, a finding not empirically established in previous myopia prevention and control studies but proposed in other public health fields (49). This notable finding suggests that peer influence continues to shape behavior into adulthood and that normative perceptions play a crucial role as predictors of myopia prevention and control behaviors. Practically, public health initiatives could harness peer influence by encouraging parent-to-parent communication, organizing community-based myopia prevention groups, and engaging schools as platforms for disseminating prevention information.

The cost of myopia prevention and control, functioning as a mooring factor within the PPM framework, was negatively associated with parents’ willingness to engage in myopia prevention and control behaviors. This finding aligns with prior research employing alternative theoretical frameworks, such as the Theory of Planned Behavior, which similarly identifies perceived cost as a barrier to health behavior intention (50, 51). Furthermore, parental willingness demonstrated a strong direct positive association with actual engagement behavior, consistent with evidence that behavioral intention is among the strongest predictors of health behavior (52, 53). Taken together, these findings suggest that financial and time costs may suppress actual parental engagement by undermining willingness, highlighting cost reduction as a priority target for intervention design.

Within the PPM framework, myopia prevention and control costs functioned as a mooring factor that negatively moderated the relationships between push/pull factors and parental willingness to engage in preventive behaviors. Specifically, higher perceived costs attenuated the influence of both health-related push factors and benefit-driven pull factors on parental willingness, suggesting that cost barriers can override motivational drivers. These findings are consistent with prior research demonstrating that economic and time-related costs constitute significant obstacles to health behavior change (54, 55). In the context of myopia prevention, where interventions often require sustained parental investment in both time and finances, including purchasing corrective devices, accompanying children to clinical visits, and supervising outdoor activities, these burdens may be particularly salient. Accordingly, policymakers should prioritize strategies that reduce families’ financial and time costs, such as subsidizing corrective eyewear, integrating screening into school-based programs, and streamlining access to ophthalmic care, thereby removing structural barriers to sustained parental engagement.

Finally, it is important to highlight that parents’ perceived value of information provision regarding myopia prevention and control policies did not positively moderate the relationship between willingness and actual engagement. This finding contradicts previous research (18) suggesting that information provision can effectively bridge the gap between intention and behavior. One potential reason for this discrepancy may be that although parents perceive myopia prevention information as valuable, such information has not been translated into actionable household-level guidance (56). Currently, these policies are predominantly driven by the education sector, with schools serving as the main implementers. This top-down approach may lead parents to perceive policy execution as sufficient, positioning them as passive observers. Although parents are primarily responsible for their children’s myopia prevention and control, their direct participation in shaping policy content has been limited. Thus, parents tend to perceive themselves more as supporters of myopia prevention policies than as active participants in their implementation. In the future, policymakers should strengthen parental engagement as an integral component of myopia prevention strategies. In particular, given that information provision alone was insufficient to bridge the willingness-action gap, future policies should move beyond information dissemination and focus on enabling parental participation through concrete measures, such as incorporating parental roles into school-based screening programs, providing structured outdoor activity plans for families, and establishing parent peer-support networks at the community level.

## Conclusion

Based on the PPM framework, this study identified key factors related to parental engagement in myopia prevention and control, thereby expanding the use of the PPM in the realm of the children and adolescents myopia prevention. To enhance the effectiveness of policy implementation and maximize its scope and impact, the government should focus on strengthening parental willingness to engage and behavior to promote myopia prevention and control at the family level. Furthermore, a better grasp of the variables affecting parental engagement in myopia prevention and control provides policymakers important information, enabling them to create focused interventions and tactics to increase parental participation and improve the overall effectiveness of myopia prevention efforts. Programs designed to stimulate parents’ willingness to engagement should focus on improving myopia risk perception and knowledge. Additionally, existing myopia prevention and control policies and campaigns should be improved. On the one hand, given the strong effect of peer influence on parents’ willingness to engage, myopia prevention campaigns should incorporate interactive, real-life scenarios that foster peer communication and mutual influence among parents. On the other hand, policies and campaigns should emphasize the central role of parents in myopia prevention and control, helping them recognize their primary responsibility and bridging the gap between intention and action.

This study has several limitations that should be acknowledged. First, the sample was drawn from the Yangtze River Delta region of China through WJX, a paid online sampling platform. Registered panel members may differ systematically from the general parenting population in their sociodemographic characteristics and health information engagement. This is particularly consequential given that several key constructs in this study may be directly related to the likelihood of panel membership, potentially resulting in an overrepresentation of health-engaged parents. Additionally, the higher proportion of female respondents (57.7%) may limit the generalizability of the findings, as parental gender may influence engagement patterns in myopia prevention. Future research should include more diverse sampling sources and consider parental gender as a potential moderating variable. Third, this study operationalized the mooring dimension using only prevention and control cost. Other contextual factors, such as competing demands from academic pressures and access to nearby eye care services, may also function as mooring forces that influence the translation of willingness into behavior. Future studies should also include more geographically and socioeconomically diverse samples and incorporate a broader set of mooring variables to provide a more comprehensive understanding of parental engagement in myopia prevention and control.

## Data Availability

The raw data supporting the conclusions of this article will be made available by the authors, without undue reservation.
